# C-Peptide Level in Fasting Plasma and Pooled Urine Predicts HbA1c after Hospitalization in Patients with Type 2 Diabetes Mellitus

**DOI:** 10.1371/journal.pone.0147303

**Published:** 2016-02-05

**Authors:** Remi Sonoda, Kentaro Tanaka, Takako Kikuchi, Yukiko Onishi, Toshiko Takao, Tazu Tahara, Yoko Yoshida, Naoki Suzawa, Shoji Kawazu, Yasuhiko Iwamoto, Akifumi Kushiyama

**Affiliations:** Division of Diabetes and Metabolism, The Institute for Adult Diseases, Asahi Life Foundation, Chuo-ku, Tokyo, Japan; National Health Research Institutes, TAIWAN

## Abstract

In this study, we investigate how measures of insulin secretion and other clinical information affect long-term glycemic control in patients with type 2 diabetes mellitus. Between October 2012 and June 2014, we monitored 202 diabetes patients who were admitted to the hospital of Asahi Life Foundation for glycemic control, as well as for training and education in diabetes management. We measured glycated hemoglobin (HbA1c) six months after discharge to assess disease management. In univariate analysis, fasting plasma C-peptide immunoreactivity (F-CPR) and pooled urine CPR (U-CPR) were significantly associated with HbA1c, in contrast to ΔCPR and C-peptide index (CPI). This association was strongly independent of most other patient variables. In exploratory factor analysis, five underlying factors, namely *insulin resistance*, *aging*, *sex differences*, *insulin secretion*, and *glycemic control*, represented patient characteristics. In particular, *insulin secretion* and *resistance* strongly influenced F-CPR, while *insulin secretion* affected U-CPR. In conclusion, the data indicate that among patients with type 2 diabetes mellitus, F-CPR and U-CPR may predict improved glycemic control six months after hospitalization.

## Introduction

Hospitalization to improve glycemic control is one of the most comprehensive short-term interventions [1] to modify lifestyle and adjust therapy [2, 3] in patients with type 2 diabetes mellitus. During this hospital stay, patients are educated and trained to prevent acute or chronic complications [4, 5], and thereby reduce future health-care costs [6]. Instruction consists of advice on diet and exercise, as well as training for insulin and/or GLP-1 self-injection [7]. However, the major goal is to improve long-term disease management.

Endogenous insulin secretion and beta cell function are crucial to diabetes management [8, 9]. In turn, insulin secretion depends on patient profile and history [10], and is typically measured using serum C-peptide levels after fasting or after an intravenous glucagon or meal tolerance test [11]. The level of endogenous insulin secretion is the basis for selecting the appropriate therapeutic agent [12, 13], which is an independent determinant of glycemic management, along with education [14].

In Japan, effective intervention for glycemic control is needed in light of escalating medical costs. In addition, identification and evaluation of variables that may predict future glycemic control could enhance the effectiveness of such educational and medical programs. Hence, we investigated patient characteristics that might be statistically associated with glycemic control after discharge, as measured by HbA1c. We hypothesized that glycemic control after discharge is correlated with clinical information at admission, including medical history, laboratory data, diabetic complications, lifestyle, and medications.

## Materials and Methods

Institutional Review Board (IRB) of the Institute for Adult Diseases, Asahi Life Foundation approved the research. The subjects gave informed consent orally, based on comprehensive written information including other studies raised in http://www.asahi-life.or.jp/pdf/hokatsudoiirai.pdf and http://www.asahi-life.or.jp/pdf/kenkyu_ichiran.pdf, to use data for this study. Data was anonymized and de-identified prior to analysis.

### Patients

Between October 2012 and June 2014, we enrolled 312 patients with type 2 diabetes mellitus who were admitted for diabetes management and education at the hospital of the Institute for Adult Diseases, Asahi Life Foundation. Admission and duration of stay were prescribed by the attending physician at an outpatient clinic. Patients had not been hospitalized at least six months prior to enrollment. We supplied missing HbA1c measurements by Last Observation Carried Forward. Thus, if measurements six months after discharge were missing, HbA1c at five months was used instead. A total of 110 patients were subsequently excluded from analysis, of whom 55 received care at a primary clinic soon after discharge. In the other 55 patients, HbA1c levels were not measured 5–6 months after discharge for various reasons. Thus, the final study population consisted of 202 patients. The study was approved by the local institutional review board, comprehensive informed consent was obtained from all patients prior to data collection, and data wasanonymized.

### Laboratory tests

The day following admission, HbA1c (Toso HLC723-G8, Tokyo), fasting plasma glucose (FPG), and fasting plasma C-peptide immune reactivity (F-CPR) were determined (Fujirebio, Tokyo). Fasting levels of uric acid (UA), estimated glomerular filtration rate (eGFR), γ-glutamyl transpeptidase (γGTP), triglyceride (TG), low-density (LDL-C), and high-density lipoprotein cholesterol (HDL-C) were also measured. In addition, postprandial CPR two hours after a meal was determined. Finally, urinary C-peptide (U-CPR) (Fujirebio, Tokyo) was measured in urine collected over 24 h, beginning at the day following admission.

### Interventions during hospitalization

Patients were provided individual guidance almost every day by physicians and certified diabetes educators. In addition, patients also received nutrition counseling two times during hospitalization from nationally registered dietitians, as well as a walk-through of menu options at each meal. Medications were adjusted by attending physicians as appropriate, and patients received medication guidance once before discharge. Finally, patients attended lectures about diabetes as a group 10 hours per week, of which five were given by physicians, and the other five by certified diabetes educators.

### Patient profiles at admission

Sex, age, diabetes duration (years living with diabetes), body mass index, hospitalizations, inpatient days, complications, and caloric restrictions were collected from medical records. HbA1c and body weight changes before admission were calculated as the difference between HbA1c and body weight on the day following admission and at one month before admission. We defined ΔCPR to be the difference between serum CPR after fasting and two hours after a meal, and we calculated C-peptide immune reactivity index (CPI) according to the formula 100 × F-CPR/FPG [15]. FPG changes and body weight changes during hospitalization were calculated as the difference between fasting plasma glucose and body weight on the day after admission and at discharge. Finally, lifestyle indicators such as smoking, alcohol use, exercise habits, cooking, living arrangements, and employment status were collected at admission.

Diabetic neuropathy was comprehensively assessed by apparent symptoms, coefficient of variation in the R-R interval (CVRR), Achilles tendon reflex (ATR), C128 tuning fork vibrator, and nerve conduction velocity (NCV). Diabetic retinopathy was assessed by funduscopy during hospitalization or within three months prior to admission. Patients were classified by ophthalmologists according to the guidelines of Japan Diabetes Society as having no diabetic retinopathy (NDR), or as having simple (SDR), pre-proliferative (PPDR), or proliferative diabetic retinopathy (PDR). Nephropathy was evaluated according to the same guidelines, and scored by estimated glomerular filtration rate and urinary albumin creatinine ratio to be in stage 1–4. Myocardial infarction or angina pectoris, as indicated by a history of coronary interventions, were considered to indicate coronary heart disease. Bleeding and ischemic strokes were noted, including lacuna infarctions confirmed by computed tomography or MRI. We measured Ankle Brachial Pressure Index (ABI) on both legs, and used the lower value.

Treatments with hypoglycemic agents, sulfonylurea (SU), biguanide (BG), glinide (GLN), α-glucosidase inhibitor (αGI), thiazolidinedione (TZD), dipeptidyl peptidase-4 inhibitor (DPP4-I), glucagon-like peptide-1 receptor agonist (GLP-1), and insulin were obtained from medical records. Lastly, we monitored adjustments in medication during hospitalization, if any.

### Statistical analysis

We used HbA1c as a measure of glycemic control, and considered the difference between HbA1c at discharge and 6 months thereafter as an indicator of long-term diabetes management. First, we performed univariate regression analyses to test the association between clinical variables at admission and HbA1c six months after discharge. Subsequently, the relationship between changes in HbA1c and indices of endogenous insulin secretion (F-CPR, U-CPR, ΔCPR, and CPI) was investigated after adjustment for other potentially confounding variables, and after stratification into tertiles. Additionally, we investigated the correlation between indices of insulin secretion and baseline HbA1c. Finally, we performed multivariate regression analysis, taking into consideration the possibility that indices of insulin secretion interact.

We then performed exploratory factor analysis and quartimin rotation to identify factors that define patient profiles. We required cumulative contribution ≥ 50% and eigenvalues ≥ 1 for the model to be adequate. Factors were then interpreted according to factor loadings on individual variables. Finally, we investigated the relationship between factors and measures of insulin secretion.

Statistical analysis was performed in JMP version 12 (SAS Institute Inc.). Statistical significance was defined at p < 0.05 for regression analysis, and at > 0.3 for factor loading. Dataset is available (S1 Dataset)

## Results

### Patient characteristics at admission

Baseline patient characteristics are summarized in Table 1. Mean HbA1c was 8.0 ± 1.4% at admission and 7.6 ± 1.2% six months after discharge.

**Table 1:** Patient characteristics, N = 202.

Variables	Value	Regression analysis against changes in HbA1c six months after discharge			
		Estimated value	SD	t value	p value
***Medical History***					
Sex (% male)	69.3	-0.560	0.18	-3.14	**0.002**
Age (years)	66.0 ± 12.0	0.022	0.01	3.13	**0.002**
Diabetes duration	19.5 ± 10.8	0.023	0.01	3.01	**0.003**
Family history of diabetes (%)	66.7	-0.070	0.18	-0.40	0.687
Body mass index (kg/m2)	25.8 ± 4.4	-0.070	0.02	-3.51	**0.001**
Hospitalizations	3 (1–9)	0.034	0.01	2.50	**0.013**
Hospital inpatient days	9.4 ± 3.6	-0.010	0.02	-0.37	0.714
Calorie intake (kcal/day)	1596 ± 198	-0.000	0.00	-4.21	**<0.0001**
***Laboratory data***					
Baseline HbA1c (%)	8.0 ± 1.4	-0.560	0.18	-3.14	**0.002**
FPG (mg/dL)	137.6 ± 38.9	-0.010	0.00	-4.96	**<0.0001**
HbA1c change before admission (%/month)	0.4 ± 0.8	0.080	0.10	0.78	0.438
Body weight change before admission (kg/month)	0.1 ± 1.2	-0.080	0.07	-1.11	0.268
F-CPR (ng/ml)	1.7 ± 1.1	-0.220	0.08	-2.73	**0.007**
CPI	1.1 (0.7–1.6)	-0.120	0.10	-1.13	0.260
ΔCPR (ng/mL)	2.3 (1.0–3.6)	-0.030	0.04	-0.81	0.421
U-CPR (μg/day)	41.6 (15–82.05)	-0.000	0.00	-2.71	**0.007**
Uric acid (mg/dL)	6.0 ± 1.4	-0.090	0.06	-1.52	0.129
γGTP (U/L)	29 (21–48.5)	-0.000	0.00	-2.17	**0.031**
triglyceride (mg/dL)	131.1 ± 68.5	-0.000	0.00	-2.26	**0.025**
HDL-C (mg/dL)	52.4 ± 15.2	0.009	0.01	1.62	0.107
LDL-C (mg/dL)	106.0 ± 28.7	-0.010	0.00	-2.13	**0.034**
***Diabetic complications***					
Neuropathy (%)	73.2 (n = 187)	0.132	0.20	0.65	0.514
CVRR (%)	2.5 ± 1.5	0.038	0.06	0.64	0.521
Ankle-Brachial Pressure Index	1.2 ± 0.1	-1.190	0.75	-1.59	0.114
Retinopathy None/Simple/PPDR/PDR (%)	49/32.7/11.4/6.9	-0.060	0.09	-0.68	0.496
Nephropathy Stage 1/2/3/4 (%)	64.4/25.2/6.9/3.4	-0.180	0.12	-1.44	0.152
Coronary heart disease (%)	25.7	0.174	0.19	0.91	0.366
Stroke (%)	17.3	0.335	0.22	1.51	0.132
***Lifestyle***					
Smoking (%)	12.6 (n = 199)	-0.380	0.26	-1.47	0.144
Drinking (%)	38.3 (n = 201)	-0.240	0.17	-1.38	0.17
Exercise (times/week)	1 (0–4)	0.021	0.03	0.63	0.526
Unemployment (%)	50.0	0.388	0.17	2.33	**0.021**
Cooking (%)	40.6	-0.120	0.17	-0.68	0.494
Living alone (%)	20.8	0.025	0.21	0.12	0.906
***Medications***					
Sulfonylurea (%)	33.7	0.048	0.09	0.54	0.592
Biguanide (%)	39.6	-0.250	0.17	-1.47	0.143
Thiazolidinedione (%)	9.9	-0.820	0.28	-2.97	**0.003**
Glinide (%)	1.0	0.573	0.70	0.82	0.411
α-glucosidase inhibitor (%)	10.9	-0.100	0.27	-0.35	0.724
DPP4 inhibitor (%)	41.6	-0.020	0.17	-0.10	0.921
Insulin (%)	50.0	-0.110	0.08	-1.30	0.195
GLP-1 receptor agonist (%)	5.9	0.677	0.35	1.92	0.057
***Changes during hospitalization***					
Body weight (kg)	1.61 ± 1.3	-0.170	0.06	-2.73	**0.007**
FPG (mg/dL)	20.9 ± 43.6	-0.010	0.00	-5.67	**< 0.0001**
Number of oral hypoglycemic agents reduced 0/1/2/3 (%)	84.7/11.4/2.5/1.5	-0.140	0.15	-0.93	0.354
Number of oral hypoglycemic agents increased 0/1/2 (%)	87.1/10.4/2.5	-0.500	0.20	-2.55	**0.012**
Biguanide increased/not changed/reduced (%)	5.4/90.6/4	-0.600	0.27	-2.21	**0.028**
TZD increased/not changed/reduced (%)	0.0/99.5/0.5	-0.980	1.20	-0.82	0.415
Sulfonylurea increased/not changed/reduced (%)	5/89.1/10.4	0.481	0.27	1.81	**0.072**
DPP4 inhibitor increased/not changed/reduced (%)	4.5/92.1/3.5	-0.410	0.30	-1.37	0.174
Glinide increased/not changed/reduced (%)	2.0/98/0.0	0.831	0.60	1.38	0.169
α-glucosidase inhibitor increased/not changed/reduced (%)	2/96.5/1.5	-0.050	0.45	-0.11	0.916
Insulin increased/not changed/reduced (%)	19.3/62.9/17.8	-0.300	0.14	-2.20	**0.029**
GLP-1 receptor agonist increased/not changed/reduced (%)	1.0/98.0/1.0	-0.770	0.60	-1.29	0.197

Data are mean ± SD or median (lower-upper quartile). Abbreviations are defined in Materials and Methods.

### Correlation between indices of insulin secretion and clinical variables

Patient characteristics at admission were tested by univariate regression for correlation with changes in HbA1c six months after discharge (Table 1). The following variables at admission have negative estimated values, and are thus interpreted as positively associated with improved HbA1c six months after discharge: sex (male), BMI, calorie intake, HbA1c, FPG, F-CPR, U-CPR, γGTP, TG, LDL-C, and TZD. Among measures of insulin secretion, F-CPR and U-CPR were significantly and positively associated with improved HbA1c six months after discharge, while CPI and ΔCPR were not. Notably, changes observed or prescribed during hospitalization were positively associated with improved HbA1c six months after discharge. These include weight loss, decreased fasting plasma glucose, and adjustments in oral hypoglycemic agents, biguanide, and insulin. On the other hand, the following variables at admission have positive estimated values, and are hence interpreted as negatively associated with improved HbA1c six months after discharge: age, hospitalizations, unemployment, diabetes duration, and changes in sulfonylurea therapy.

**Fig 1:**
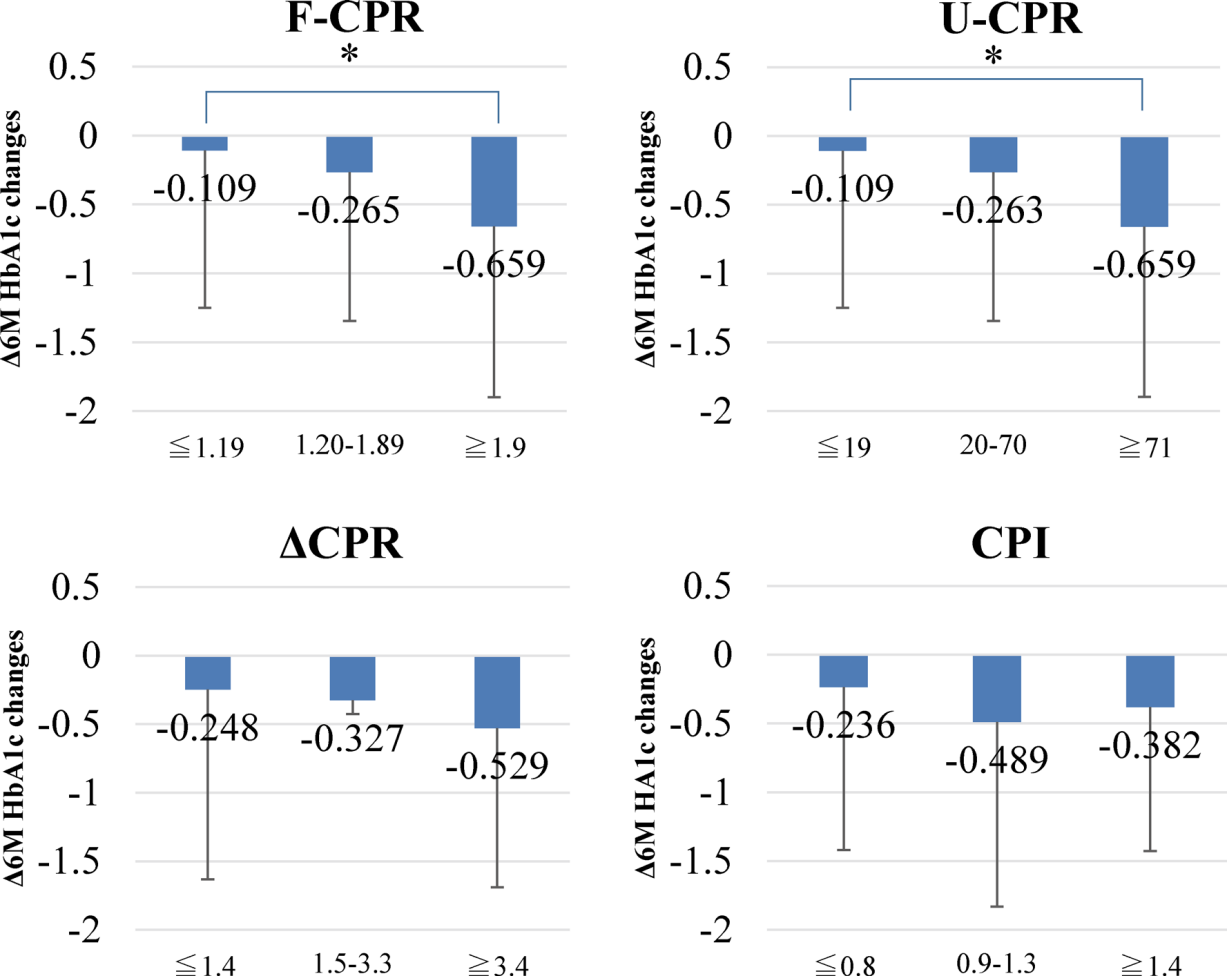
Relationship between HbA1c changes and four indices of insulin secretion. Subjects were stratified by the tertiles of four indices of insulin secretion such as F-CPR, U-CPR, ΔCPR, and CPI, and HbA1c changes were compared high and mid tertiles with lowest insulinogenic tertile as standard. *, p < 0.05 by ANOVA and post-hoc Dunnett’s test.

Further, we investigated the relationship between HbA1c changes and F-CPR, U-CPR, ΔCPR, and CPI stratified into tertiles (Fig 1). Patients with the highest tertiles of F-CPR and U-CPR experienced a significantly larger decrease than those with the lowest in HbA1c change.

Subsequently, multivariate analyses were performed to test whether the relationship between indices of insulin secretion and changes in HbA1c is confounded by other variables (Table 2 and S1 Table). Both F-CPR and U-CPR were independent of most variables except diabetes duration. However, F-CPR was dependent on body mass index, while U-CPR was dependent on the change in fasting plasma glucose during hospitalization. In contrast, ΔCPR was dependent on most variables except baseline HbA1c. Similarly, CPI was dependent on most variables except baseline HbA1c and F-CPR.

**Table 2:** Relationship between HbA1c changes at six months and insulin secretion indices, F-CPR, U-CPR, ΔCPR and CPI by regression analysis.

	F-CPR	U-CPR	ΔCPR	CPI
	adjusted by	adjusted by	adjusted by	adjusted by
None	✔	✔		
Sex	✔	✔		
Age	✔	✔		
Diabetes duration				
Family history of diabetes	✔	✔		
Body mass index		✔		
Hospitalizations	✔	✔		
Hospital inpatient days	✔	✔		
Calorie intake	✔	✔		
Baseline HbA1c	✔	✔	✔	✔
FPG	✔	✔		
HbA1c change before admission	✔	✔		
Body weight change before admission	✔	✔		
F-CPR		✔		✔
U-CPR				
ΔCPR	✔	✔		
CPI	✔	✔		
Uric acid	✔	✔		
γGTP	✔	✔		
Triglyceride	✔	✔		
HDL-C	✔	✔		
LDL-C	✔	✔		
Neuropathy	✔	✔		
CVRR	✔	✔		
Retinopathy	✔	✔		
Nephropathy	✔	✔		
Coronary heart disease	✔	✔		
Stroke	✔	✔		
Ankle-Brachial Pressure Index	✔	✔		
Smoking	✔	✔		
Drinking	✔	✔		
Exercise	✔	✔		
Unemployment	✔	✔		
Cooking	✔	✔		
Living alone	✔	✔		
Sulfonylurea	✔	✔		
Biguanide	✔	✔		
Thiazolidinedione	✔	✔		
Glinide	✔	✔		
α-glucosidase inhibitor	✔	✔		
DPP4 inhibitor	✔	✔		
Insulin	✔	✔		
GLP-1 receptor agonist	✔	✔		
Body weight change during hospitalization	✔	✔		
FPG change during hospitalization	✔			
Number of OHA reduced	✔	✔		
Number of OHA increased	✔	✔		
Change in biguanide	✔	✔		
Change in thiazolidinedione	✔	✔		
Change in sulfonylurea	✔	✔		
Change in DPP4 inhibitor	✔	✔		
Change in glinide	✔	✔		
Change in α-glucosidase inhibitor	✔	✔		
Change in insulin	✔	✔		
Change in GLP-1 receptor agonist	✔	✔		

p values are < 0.05 in circled variables. Abbreviations are defined in Materials and Methods.

**Fig 2:**
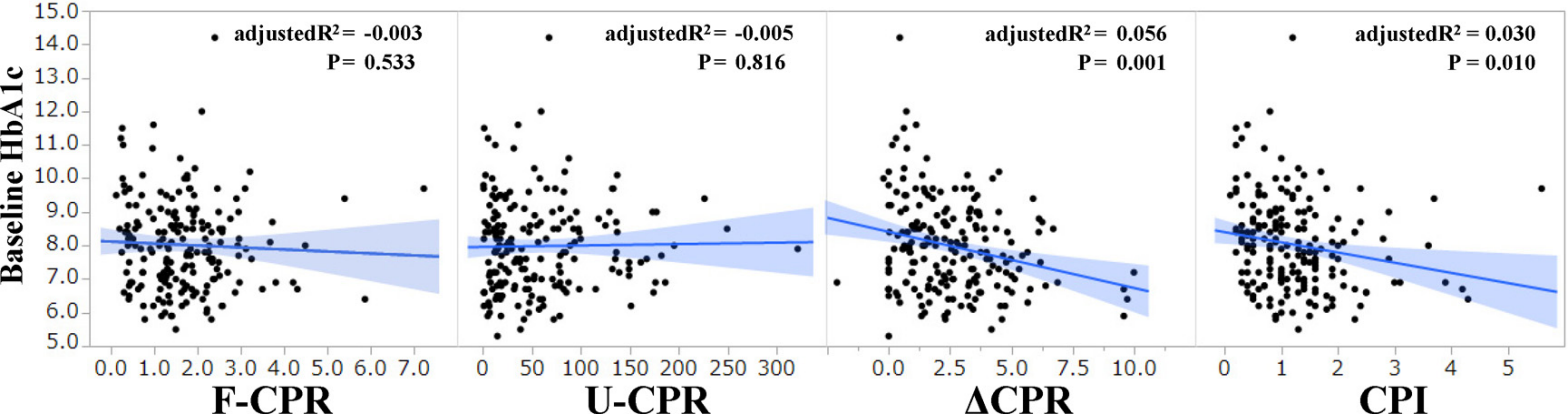
Relationship between baseline HbA1c and indices of insulin secretion. Scatter plots were shown for the relationships between baseline HbA1c and four indices of insulin secretion such as F-CPR, U-CPR, ΔCPR and CPI. Regression lines, adjusted R^2^ and p value were also shown in the plots.

Furthermore, ΔCPR (p = 0.001) and CPI (p = 0.01) were positively associated with baseline HbA1c, while F-CPR and U-CPR were not (Fig 2).

To investigate the relationship between F-CPR and U-CPR more closely, multivariate regression analysis was performed (Table 3). This analysis indicated that U-CPR and U-CPR×F-CPR were significantly associated with changes in HbA1c after discharge, while F-CPR was not significant when interaction was considered. Based on this analysis, HbA1c changes after discharge maybe predicted according to the equation 0.045 + (-0.006 × U-CPR) + (-0.088 × F-CPR) + (U-CPR-57.492) ×((F-CPR—1.669) × 0.004). Using both F-CPR and U-CPR would enhance prediction accuracy, although F-CPR is easier to obtain than U-CPR in outpatient settings.

**Table 3:** Multivariate regression analysis for HbA1c changes six months after discharge using U-CPR, F-CPR, and U-CPR×F-CPR.

Index	Estimated value	SD	t value	p value
U-CPR	-0.006	0.002	-2.94	**0.004**
F-CPR	-0.088	0.090	-0.97	0.331
U-CPR×F-CPR	0.004	0.001	2.67	**0.008**

Underlined p values are < 0.05. Abbreviations are defined in Materials and Methods.

### Exploratory factor analysis

We hypothesized that five underlying factors at admission, such as *medical history*, *laboratory data*, *diabetic complications*, *lifestyle*, and *medications* (Table 1), may reasonably define patient profiles. Thus, we modeled patient data using five factors, and model fit statistics and loading values were examined after oblique quartimin rotation. Variables with communality < 0.3 after factor extraction were excluded in subsequent rounds of analysis. Finally, 21 variables remained with communality > 0.3 (Table 4) and cumulative contribution 55.5%. The final model contained these 21 variables in five factors, at which the eigenvalue was > 1 on a scree plot. Thus, we concluded that a model with five factors was statistically appropriate, and incidentally coincided with our hypothesis.

**Table 4:** Factor loadings on patient characteristics.

	Insulin resistance	Aging	Sex differences	Insulin secretion	Glycemic control
CPI	**0.607**	-0.021	-0.161	**0.677**	**-0.389**
F-CPR	**0.570**	0.001	-0.126	**0.768**	-0.057
Body mass index	**0.562**	-0.230	-0.085	0.081	0.175
Uric acid	**0.461**	-0.011	0.185	0.069	0.017
Nephropathy	**0.444**	0.194	0.018	-0.212	0.051
Retinopathy	**0.301**	0.170	0.010	**-0.454**	0.238
Age	-0.223	**0.821**	-0.139	0.083	-0.132
Diabetes duration	0.003	**0.632**	0.117	**-0.355**	-0.100
Exercise	0.079	**0.484**	0.027	-0.240	-0.132
CVRR	-0.027	**-0.407**	-0.088	-0.050	-0.154
Coronary heart disease	0.207	**0.455**	-0.026	-0.019	0.051
Unemployment	-0.168	**0.500**	-0.185	0.014	-0.071
Sex	0.050	0.026	**1.007**	0.034	-0.095
Calorie intake	0.104	-0.249	**0.713**	0.063	0.023
Cooking	-0.073	0.075	**0.560**	-0.009	-0.016
ΔCPR	0.086	-0.123	0.000	**0.476**	-0.130
U-CPR	-0.018	-0.111	0.091	**0.548**	0.085
Sulfonylurea	-0.010	0.105	0.029	**0.424**	0.160
Insulin	0.095	0.087	-0.015	**-0.642**	0.056
FPG	-0.078	0.031	-0.005	0.258	**0.918**
Baseline HbA1c	0.140	-0.088	-0.037	-0.176	**0.494**

Factor loadings > 0.300 were considered significant and are underlined. Abbreviations are defined in Materials and Methods.

Factor loadings are listed in Table 4, in which loadings > 0.3 are considered significant and are underlined. Factors were interpreted according to the clinical features of variables that were significantly affected. Hence, CPI, F-CPR, body mass index, uric acid, nephropathy, and retinopathy were considered indicators of *insulin resistance*. On the other hand, age, diabetes duration, hospitalizations, CVRR, coronary heart disease, and unemployment were considered indicators of *aging*. The factor *sex differences* included the variables sex, calorie intake, and cooking, while *insulin secretion* contained the variables CPI, F-CPR, retinopathy, diabetes duration, ΔCPR, U-CPR, sulfonylurea, and insulin treatment. Finally, indicators of *glycemic control* included CPI, fasting plasma glucose, and HbA1c.

**Fig 3:**
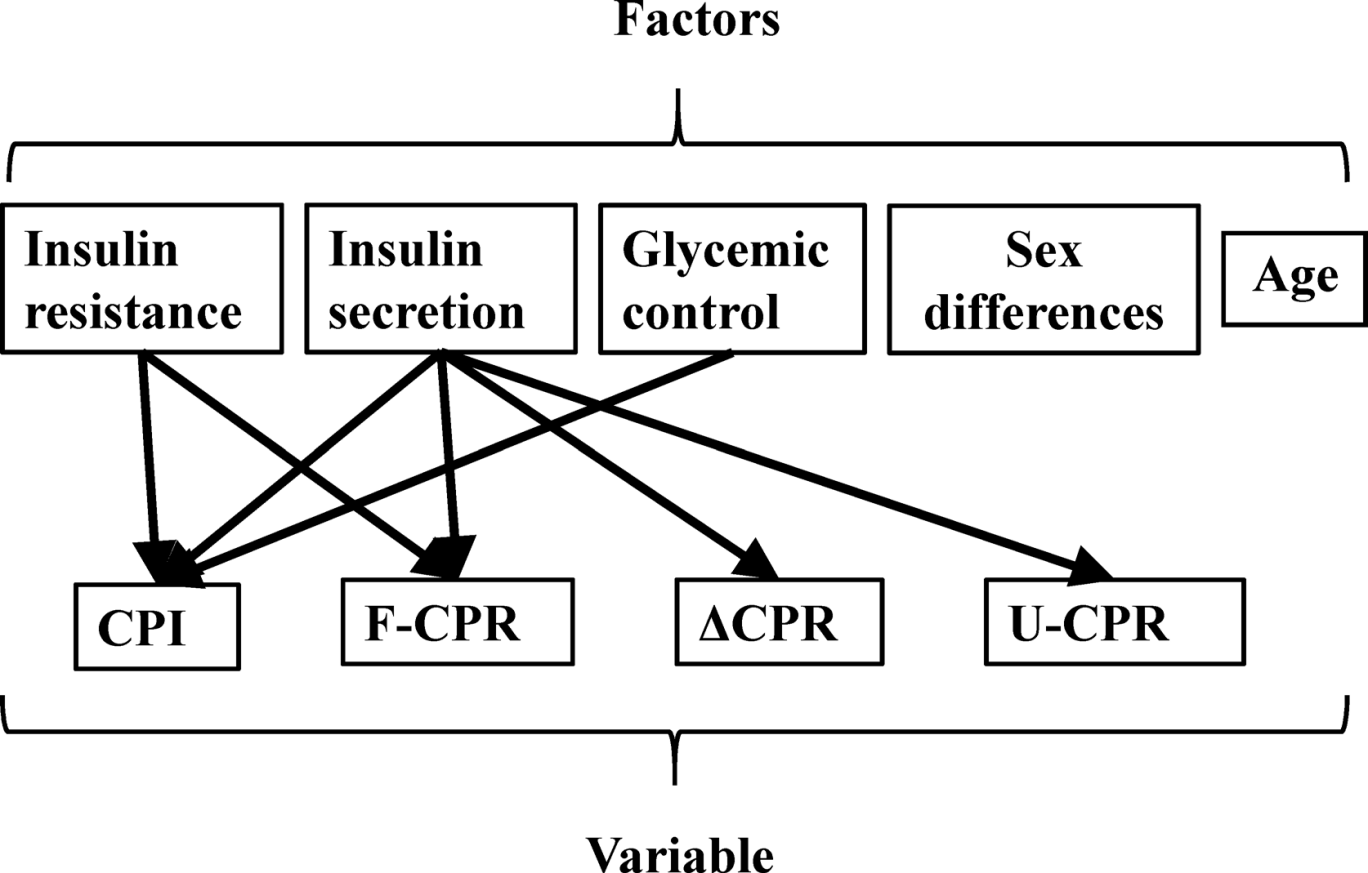
Relationship between factors and variables related to insulin secretion. The effect of factors on CPI, F-CPR, U-CPR, and ΔCPR, which are primarily indicators of *insulin secretion*.

Notably, while CPI, F-CPR, U-CPR, and ΔCPR are indices of *insulin secretion*, these variables were also affected by other factors (Fig 3). For example, F-CPR and CPI were also controlled by *insulin resistance*, and *glycemic control* additionally influenced CPI.

## Discussion

Prediction of glycemic control after hospital-based intervention for diabetes management is rarely reported, and has only been examined in relation to basic data such as age, gender, body mass index, HbA1c, and blood glucose [16]. Unfortunately, many reports investigate such interventions as an endpoint or a self-contained event in managing diabetes [17, 18]. In this paper, we analyzed patient profiles comprehensively to identify factors and variables that predict sustained glycemic control after discharge. Of note, glycated albumin at discharge was found to be a useful marker of deteriorating glycemic control after discharge [19].

We first found 21 variables from numerous variables, including patient characteristics as well as observed changes and prescribed therapy adjustments during hospitalization. However, we reasoned that confounding effects were likely to be present because of the large number of variables. Of these variables, F-CPR and U-CPR were found to be significant predictors of changes in HbA1c, and mostly remain so after adjustment for other variables. Naturally, body mass index and diabetes duration, which are major indices of beta cell function in patients with type 2 diabetes [20], confounded the effects of these variables. On the other hand, HbA1c was not significantly associated with CPI and ΔCPR. Presumably, CPI is insignificant only because it is the ratio of F-CPR to fasting plasma glucose, which are both positively associated with HbA1c.

There was a previous document that postprandial CPR index was significantly associated with achievement of HbA1c⋖7.0% [21]. There are many differences from our data in patients’ background, especially very high baseline HbA1c and different evaluation endpoints. The report insists the predictive performance of the various indices of insulin secretion were similar, although they hardly refer to confounding and interaction among the indices and variables. Our comprehensive analysis clarified whether ΔCPR and CPR index can predict or not is own to subjects’ background or profile, and F-CPR and U-CPR are considerably independent.

There are some studies for prediction of other endpoints or other situations by these indices. Future insulin use was predicted by BMI, FPG, F-CPR and U-CPR [22], and F-CPR was associated with glycemic control after bariatric surgery [23]. Glucagon-stimulated ΔCPR predicts the efficacy of GLP-1 [24]. Although there might be commonality in the predictive performance of F-CPR and U-CPR in various treatment for glycemic control, further study is necessary to clarify the specific aspects of these indices for the effect of hospitalization and/or other treatments.

We also performed factor analysis to identify factors that characterize patients. We detected five such factors, which aggregated patient characteristics very differently from the initial, ‘natural’ classification of variables in Table 1. Remarkably, the relationship between diabetes management and some patient characteristics are clarified by factor analysis. For example, unemployment is associated with the factor *aging* probably due to retirement. In addition, cooking is influenced by the factor *sex differences* because females are traditionally the primary cook in most Japanese families.

On the other hand, diabetes duration and body mass index are influenced by the factors *insulin secretion* and *insulin resistance*, respectively. Thus, both factors also affect F-CPR, which is correlated with body mass index and diabetes duration, according to multivariate regression. Similarly, U-CPR depends on the factor *insulin secretion*, because it is also associated with diabetes duration.

Endogenous insulin secretion can be measured by several indices [15, 25–28], including immune-reactive insulin, homeostatic model assessment of beta cell function, insulinogenic index, glucagon test, CPI, and U-CPR. In practical terms, however, F-CPR is the most convenient variable to use in outpatient settings, as it requires a single blood sample, whereas U-CPR requires urine collection over 24 hours.

Our study has several limitations. First, we did not consider differences in therapeutic goals, which, in Japan, are individually set based on age, diabetes duration, complications, social support, and hypoglycemia [29, 30]. In addition, this study was retrospective in nature and was conducted in one hospital with specific protocols for hospitalization, educational intervention, and selection of hypoglycemic agents. It is also likely that patient profiles will vary across communities, countries, and races. Socioeconomic disparities are often observed as well in access to hospital-based programs [31]. Finally, the impact of hospital-based education on patients should also be evaluated. For instance, educational impact may differ even in the same patient depending on the number of hospitalizations. Indeed, our data indicate that fewer hospitalizations tend to result in better HbA1c levels (Table 1). In addition, educational impact may also be influenced by socioeconomic status [32], even though social variables such as aging and sex do not confound the effects of F-CPR and U-CPR.

Nevertheless, F-CPR and U-CPR are strongly independent of most clinical variables. Thus, measuring C-peptide might be useful as a marker to predict whether existing hospitalization-based educational programs would be effective in long-term glycemic control, or whether additional, recurrent medical interventions would be needed. In the concrete, when the predictive result and target HbA1c are compared, physician can became conscious and modify strategy for the glycemic control. Our results will also be applied to refine intervention program during hospitalization. It is possible to target patients with difficulty in glycemic control for active medical intervention besides standard program, during hospitalization or immediately after discharge to keep good glycemic control after hospitalization.

Our results also indicate that factor analysis can distill complex clinical information into a few explanatory factors. In particular, it is worth investigating whether aggregating factors such as *insulin resistance* and *secretion* as defined here are universally associated with diabetes management after discharge.

## Conclusion

In patients with type 2 diabetes mellitus, F-CPR and U-CPR predict improved glycemic control six months after hospitalization for diabetes management.

## Supporting Information

S1 Dataset (JMP)

S1 Tables Regression analysis between HbA1c changes at six months and insulin secretion indices adjusted by other variables. (DOCX)
