# Supplementary material for: C-Peptide Level in Fasting Plasma and Pooled Urine Predicts HbA1c after Hospitalization in Patients with Type 2 Diabetes Mellitus
Source: PLoS One. 2016 Feb 5;11(2):e0147303. doi: 10.1371/journal.pone.0147303 (PMC4743946; doi:10.1371/journal.pone.0147303)
Supplement: S1 Tables — (DOCX) [file pone.0147303.s002.docx]

S1 Tables. Relationships between HbA1c changes at six months and insulin secretion indices by regression analysis adjusted by other variables.

Regression analysis for HbA1c changes explained by F-CPR adjusted by another variable.

| **F-CPR adjusted by** | **Estimated value** | **SD** | **t value** | **p value** |
| --- | --- | --- | --- | --- |
| None | -0.221 | 0.081 | -2.73 | 0.007 |
| Sex | -0.231 | 0.079 | -2.92 | 0.004 |
| Age | -0.178 | 0.083 | -2.14 | 0.033 |
| Diabetes duration | -0.149 | 0.090 | -1.65 | 0.100 |
| Family history of diabetes | -0.237 | 0.083 | -2.86 | 0.005 |
| Body mass index | -0.132 | 0.090 | -1.46 | 0.146 |
| Hospitalizations | -0.185 | 0.083 | -2.21 | 0.028 |
| Hospital inpatient days | -0.226 | 0.081 | -2.79 | 0.006 |
| Calorie intake | -0.189 | 0.079 | -2.41 | 0.017 |
| Baseline HbA1c | -0.249 | 0.067 | -3.73 | 0.000 |
| FPG | -0.175 | 0.079 | -2.22 | 0.028 |
| HbA1c change before admission | -0.222 | 0.081 | -2.74 | 0.007 |
| Body weight change before admission | -0.232 | 0.081 | -2.87 | 0.005 |
| CPI | -0.928 | 0.205 | -4.53 | < .0001 |
| ΔCPR | -0.245 | 0.091 | -2.69 | 0.008 |
| U-CPR | -0.151 | 0.088 | -1.71 | 0.089 |
| Uric acid | -0.199 | 0.085 | -2.33 | 0.021 |
| γGTP | -0.189 | 0.082 | -2.29 | 0.023 |
| triglyceride | -0.178 | 0.086 | -2.07 | 0.040 |
| HDL-C | -0.206 | 0.084 | -2.46 | 0.015 |
| LDL-C | -0.216 | 0.080 | -2.69 | 0.008 |
| Neuropathy | -0.223 | 0.085 | -2.62 | 0.010 |
| CVRR | -0.250 | 0.085 | -2.92 | 0.004 |
| Retinopathy | -0.251 | 0.083 | -3.04 | 0.003 |
| Nephropathy | -0.217 | 0.081 | -2.70 | 0.008 |
| Coronary heart disease | -0.220 | 0.081 | -2.72 | 0.007 |
| Stroke | -0.219 | 0.080 | -2.72 | 0.007 |
| Ankle-Brachial Pressure Index | -0.216 | 0.080 | -2.69 | 0.008 |
| Smoking | -0.212 | 0.082 | -2.59 | 0.010 |
| Drinking | -0.217 | 0.081 | -2.69 | 0.008 |
| Exercise | -0.206 | 0.082 | -2.51 | 0.013 |
| Unemployment | -0.201 | 0.082 | -2.45 | 0.015 |
| Cooking | -0.231 | 0.081 | -2.85 | 0.005 |
| Living alone | -0.218 | 0.081 | -2.69 | 0.008 |
| Sulfonylurea | -0.233 | 0.085 | -2.74 | 0.007 |
| Biguanide | -0.210 | 0.082 | -2.56 | 0.011 |
| Thiazolidinedione | -0.208 | 0.079 | -2.63 | 0.009 |
| Glinide | -0.218 | 0.081 | -2.70 | 0.008 |
| α-glucosidase inhibitor | -0.223 | 0.081 | -2.74 | 0.007 |
| DPP4 inhibitor | -0.221 | 0.081 | -2.73 | 0.007 |
| Insulin | -0.237 | 0.092 | -2.58 | 0.011 |
| GLP-1 receptor agonist | -0.242 | 0.081 | -2.99 | 0.003 |
| Body weight change during hospitalization | -0.186 | 0.081 | -2.28 | 0.024 |
| FPG change during hospitalization | -0.170 | 0.077 | -2.22 | 0.028 |
| Number of OHA reduced | -0.219 | 0.081 | -2.69 | 0.008 |
| Number of OHA increased | -0.199 | 0.081 | -2.46 | 0.015 |
| Change in biguanide | -0.200 | 0.081 | -2.46 | 0.015 |
| Change in thiazolidinedione | -0.219 | 0.081 | -2.71 | 0.007 |
| Change in sulfonylurea | -0.203 | 0.082 | -2.46 | 0.015 |
| Change in DPP4 inhibitor | -0.207 | 0.081 | -2.56 | 0.011 |
| Change in glinide | -0.229 | 0.081 | -2.85 | 0.005 |
| Change in α-glucosidase inhibitor | -0.222 | 0.081 | -2.74 | 0.007 |
| Change in insulin | -0.206 | 0.081 | -2.53 | 0.012 |
| Change in GLP-1 receptor agonist | -0.225 | 0.081 | -2.80 | 0.006 |

Underlined p values are < 0.05. Abbreviations are defined in Materials and Methods.

Regression analysis for HbA1c changes explained by U-CPR adjusted by another variable.

| **U-CPR adjusted by** | **Estimated value** | **SD** | **t value** | **p value** |
| --- | --- | --- | --- | --- |
| None | -0.004 | 0.002 | -2.71 | 0.007 |
| Sex | -0.004 | 0.002 | -2.43 | 0.016 |
| Age | -0.003 | 0.002 | -2.11 | 0.036 |
| Diabetes duration | -0.003 | 0.002 | -1.73 | 0.086 |
| Family history of diabetes | -0.004 | 0.002 | -2.66 | 0.009 |
| Body mass index | -0.003 | 0.002 | -2.12 | 0.035 |
| Hospitalizations | -0.003 | 0.002 | -2.13 | 0.035 |
| Hospital inpatient days | -0.004 | 0.002 | -2.71 | 0.007 |
| Calorie intake | -0.003 | 0.002 | -2.12 | 0.036 |
| HbA1c | -0.004 | 0.001 | -3.05 | 0.003 |
| FPG | -0.003 | 0.002 | -2.05 | 0.042 |
| HbA1c change before admission | -0.004 | 0.002 | -2.67 | 0.008 |
| Body weight change before admission | -0.004 | 0.002 | -2.72 | 0.007 |
| CPI | -0.004 | 0.002 | -2.45 | 0.015 |
| ΔCPR | -0.004 | 0.002 | -2.45 | 0.015 |
| F-CPR | -0.003 | 0.002 | -1.69 | 0.093 |
| Uric acid | -0.004 | 0.002 | -2.79 | 0.006 |
| γGTP | -0.004 | 0.002 | -2.68 | 0.008 |
| triglyceride | -0.004 | 0.002 | -2.40 | 0.018 |
| HDL-C | -0.004 | 0.002 | -2.57 | 0.011 |
| LDL-C | -0.004 | 0.002 | -2.46 | 0.015 |
| Neuropathy | -0.004 | 0.002 | -2.43 | 0.016 |
| CVRR | -0.004 | 0.002 | -2.52 | 0.013 |
| Retinopathy | -0.004 | 0.002 | -2.63 | 0.009 |
| Nephropathy | -0.005 | 0.002 | -3.01 | 0.003 |
| Coronary heart disease | -0.004 | 0.002 | -2.88 | 0.004 |
| Stroke | -0.004 | 0.002 | -2.62 | 0.010 |
| Ankle-Brachial Pressure Index | -0.004 | 0.002 | -2.63 | 0.009 |
| Smoking | -0.004 | 0.002 | -2.58 | 0.011 |
| Drinking | -0.004 | 0.002 | -2.52 | 0.013 |
| Exercise | -0.004 | 0.002 | -2.45 | 0.015 |
| Unemployment | -0.004 | 0.002 | -2.47 | 0.015 |
| Cooking | -0.004 | 0.002 | -2.69 | 0.008 |
| Living alone | -0.004 | 0.002 | -2.74 | 0.007 |
| Sulfonylurea | -0.004 | 0.002 | -2.71 | 0.007 |
| Biguanide | -0.004 | 0.002 | -2.67 | 0.008 |
| Thiazolidinedione | -0.004 | 0.002 | -2.62 | 0.009 |
| Glinide | -0.004 | 0.002 | -2.73 | 0.007 |
| α-glucosidase inhibitor | -0.004 | 0.002 | -2.70 | 0.008 |
| DPP4 inhibitor | -0.004 | 0.002 | -2.77 | 0.006 |
| Insulin | -0.005 | 0.002 | -2.73 | 0.007 |
| GLP-1 receptor agonist | -0.004 | 0.002 | -2.90 | 0.004 |
| Body weight change during hospitalization | -0.004 | 0.002 | -2.36 | 0.019 |
| FPG change during hospitalization | -0.003 | 0.001 | -1.86 | 0.064 |
| Number of OHA reduced | -0.004 | 0.002 | -2.69 | 0.008 |
| Number of OHA increased | -0.003 | 0.002 | -2.23 | 0.027 |
| Change in biguanide | -0.004 | 0.002 | -2.35 | 0.020 |
| Change in thiazolidinedione | -0.004 | 0.002 | -2.71 | 0.007 |
| Change in sulfonylurea | -0.004 | 0.002 | -2.62 | 0.010 |
| Change in DPP4 inhibitor | -0.004 | 0.002 | -2.55 | 0.012 |
| Change in glinide | -0.004 | 0.002 | -2.79 | 0.006 |
| Change in α-glucosidase inhibitor | -0.004 | 0.002 | -2.67 | 0.008 |
| Change in insulin | -0.004 | 0.002 | -2.58 | 0.011 |
| Change in GLP-1 receptor agonist | -0.004 | 0.002 | -2.65 | 0.009 |

Underlined p values are < 0.05. Abbreviations are defined in Materials and Methods.

Regression analysis for HbA1c changes explained by ΔCPR adjusted by another variable.

| **ΔCPR adjusted by** | **Estimated value** | **SD** | **t value** | **p value** |
| --- | --- | --- | --- | --- |
| None | -0.034 | 0.042 | -0.81 | 0.421 |
| Sex | -0.032 | 0.042 | -0.76 | 0.447 |
| Age | -0.010 | 0.042 | -0.24 | 0.809 |
| Diabetes duration | 0.004 | 0.045 | 0.09 | 0.926 |
| Family history of diabetes | -0.038 | 0.044 | -0.86 | 0.389 |
| Body mass index | -0.003 | 0.042 | -0.08 | 0.936 |
| Hospitalizations | 0.005 | 0.044 | 0.11 | 0.914 |
| Hospital inpatient days | -0.035 | 0.042 | -0.83 | 0.409 |
| Calorie intake | -0.018 | 0.041 | -0.45 | 0.655 |
| Baseline HbA1c | -0.121 | 0.036 | -3.41 | 0.001 |
| FPG | -0.034 | 0.040 | -0.85 | 0.396 |
| HbA1c change before admission | -0.032 | 0.043 | -0.74 | 0.457 |
| Body weight change before admission | -0.034 | 0.042 | -0.81 | 0.417 |
| CPI | -0.012 | 0.051 | -0.23 | 0.815 |
| F-CPR | 0.029 | 0.050 | 0.58 | 0.561 |
| U-CPR | -0.016 | 0.045 | -0.35 | 0.728 |
| Uric acid | -0.026 | 0.043 | -0.59 | 0.555 |
| γGTP | -0.021 | 0.043 | -0.49 | 0.624 |
| triglyceride | -0.038 | 0.042 | -0.91 | 0.366 |
| HDL-C | -0.026 | 0.043 | -0.60 | 0.551 |
| LDL-C | -0.036 | 0.042 | -0.86 | 0.389 |
| Neuropathy | -0.024 | 0.045 | -0.53 | 0.599 |
| CVRR | -0.036 | 0.044 | -0.81 | 0.419 |
| Retinopathy | -0.051 | 0.045 | -1.15 | 0.254 |
| Nephropathy | -0.044 | 0.043 | -1.02 | 0.308 |
| Coronary heart disease | -0.031 | 0.042 | -0.73 | 0.467 |
| Stroke | -0.033 | 0.042 | -0.79 | 0.429 |
| Ankle-Brachial Pressure Index | -0.027 | 0.042 | -0.65 | 0.519 |
| Smoking | -0.027 | 0.043 | -0.63 | 0.529 |
| Drinking | -0.030 | 0.042 | -0.71 | 0.480 |
| Exercise | -0.033 | 0.042 | -0.77 | 0.444 |
| Unemployment | -0.027 | 0.042 | -0.63 | 0.526 |
| Cooking | -0.037 | 0.043 | -0.87 | 0.385 |
| Living alone | -0.035 | 0.043 | -0.82 | 0.411 |
| Sulfonylurea | -0.031 | 0.043 | -0.73 | 0.466 |
| Biguanide | -0.028 | 0.042 | -0.65 | 0.517 |
| Thiazolidinedione | -0.023 | 0.042 | -0.54 | 0.590 |
| Glinide | -0.038 | 0.042 | -0.89 | 0.377 |
| α-glucosidase inhibitor | -0.033 | 0.042 | -0.79 | 0.432 |
| DPP4 inhibitor | -0.034 | 0.043 | -0.79 | 0.428 |
| Insulin | -0.018 | 0.047 | -0.39 | 0.697 |
| GLP-1 receptor agonist | -0.031 | 0.042 | -0.73 | 0.466 |
| Body weight change during hospitalization | -0.013 | 0.043 | -0.31 | 0.759 |
| FPG change during hospitalization | -0.020 | 0.040 | -0.49 | 0.622 |
| Number of OHA reduced | -0.042 | 0.043 | -0.96 | 0.336 |
| Number of OHA increased | -0.024 | 0.041 | -0.59 | 0.554 |
| Change in biguanide | -0.031 | 0.041 | -0.75 | 0.454 |
| Change in thiazolidinedione | -0.034 | 0.042 | -0.80 | 0.426 |
| Change in sulfonylurea | -0.035 | 0.042 | -0.83 | 0.406 |
| Change in DPP4 inhibitor | 0.017 | 0.062 | 0.28 | 0.782 |
| Change in glinide | -0.038 | 0.042 | -0.90 | 0.368 |
| Change in α-glucosidase inhibitor | -0.034 | 0.042 | -0.79 | 0.429 |
| Change in insulin | -0.028 | 0.045 | -0.63 | 0.530 |
| Change in GLP-1 receptor agonist | -0.037 | 0.042 | -0.87 | 0.384 |

Underlined p values are < 0.05. Abbreviations are defined in Materials and Methods.

Regression analysis for HbA1c changes explained by CPI adjusted by another variable.

| **CPI adjusted by** | **Estimated value** | **SD** | **t value** | **p value** |
| --- | --- | --- | --- | --- |
| None | -0.118 | 0.104 | -1.13 | 0.260 |
| Sex | -0.147 | 0.102 | -1.43 | 0.153 |
| Age | -0.067 | 0.105 | -0.64 | 0.524 |
| Diabetes duration | -0.002 | 0.113 | -0.02 | 0.988 |
| Family history of diabetes | -0.122 | 0.107 | -1.15 | 0.253 |
| Body mass index | 0.007 | 0.111 | 0.07 | 0.947 |
| Hospitalizations | -0.071 | 0.106 | -0.67 | 0.505 |
| Hospital inpatient days | -0.124 | 0.104 | -1.19 | 0.236 |
| Calorie intake | -0.104 | 0.100 | -1.04 | 0.300 |
| Baseline HbA1c | -0.272 | 0.087 | -3.12 | 0.002 |
| FPG | -0.185 | 0.100 | -1.86 | 0.065 |
| HbA1c change before admission | -0.120 | 0.104 | -1.15 | 0.250 |
| Body weight change before admission | -0.129 | 0.104 | -1.23 | 0.219 |
| ΔCPR | -0.105 | 0.118 | -0.88 | 0.378 |
| F-CPR | 0.970 | 0.260 | 3.74 | 0.000 |
| U-CPR | -0.023 | 0.109 | -0.21 | 0.835 |
| Uric acid | -0.076 | 0.109 | -0.70 | 0.484 |
| γGTP | -0.088 | 0.104 | -0.85 | 0.398 |
| triglyceride | -0.132 | 0.104 | -1.27 | 0.206 |
| HDL-C | -0.089 | 0.107 | -0.83 | 0.410 |
| LDL-C | -0.124 | 0.103 | -1.20 | 0.232 |
| Neuropathy | -0.108 | 0.110 | -0.98 | 0.329 |
| CVRR | -0.174 | 0.109 | -1.59 | 0.113 |
| Retinopathy | -0.148 | 0.109 | -1.36 | 0.175 |
| Nephropathy | -0.114 | 0.104 | -1.10 | 0.273 |
| Coronary heart disease | -0.116 | 0.104 | -1.11 | 0.269 |
| Stroke | -0.125 | 0.104 | -1.20 | 0.231 |
| Ankle-Brachial Pressure Index | -0.115 | 0.103 | -1.11 | 0.267 |
| Smoking | -0.105 | 0.105 | -0.99 | 0.322 |
| Drinking | -0.120 | 0.104 | -1.15 | 0.250 |
| Exercise | -0.115 | 0.104 | -1.10 | 0.272 |
| Unemployment | -0.092 | 0.105 | -0.88 | 0.382 |
| Cooking | -0.134 | 0.105 | -1.27 | 0.206 |
| Living alone | -0.113 | 0.105 | -1.08 | 0.284 |
| Sulfonylurea | -0.115 | 0.107 | -1.07 | 0.284 |
| Biguanide | -0.104 | 0.105 | -0.99 | 0.321 |
| Thiazolidinedione | -0.120 | 0.102 | -1.18 | 0.239 |
| Glinide | -0.115 | 0.104 | -1.10 | 0.273 |
| α-glucosidase inhibitor | -0.119 | 0.105 | -1.13 | 0.260 |
| DPP4 inhibitor | -0.117 | 0.104 | -1.12 | 0.262 |
| Insulin | -0.092 | 0.117 | -0.78 | 0.435 |
| GLP-1 receptor agonist | -0.141 | 0.105 | -1.34 | 0.181 |
| Body weight change during hospitalization | -0.080 | 0.103 | -0.78 | 0.438 |
| FPG change during hospitalization | -0.161 | 0.097 | -1.66 | 0.099 |
| Number of OHA reduced | -0.106 | 0.108 | -0.98 | 0.327 |
| Number of OHA increased | -0.081 | 0.103 | -0.79 | 0.431 |
| Change in biguanide | -0.153 | 0.146 | -1.05 | 0.298 |
| Change in thiazolidinedione | 0.418 | 0.491 | 0.85 | 0.406 |
| Change in sulfonylurea | -0.023 | 0.174 | -0.13 | 0.896 |
| Change in DPP4 inhibitor | -0.169 | 0.182 | -0.93 | 0.356 |
| Change in glinide | -0.131 | 0.104 | -1.26 | 0.209 |
| Change in α-glucosidase inhibitor | -0.265 | 0.243 | -1.09 | 0.289 |
| Change in insulin | -0.136 | 0.111 | -1.22 | 0.224 |
| Change in GLP-1 receptor agonist | -0.132 | 0.104 | -1.27 | 0.207 |

Underlined p values are < 0.05. Abbreviations are defined in Materials and Methods.
